# The Placental Innate Immune System Is Altered in Early-Onset Preeclampsia, but Not in Late-Onset Preeclampsia

**DOI:** 10.3389/fimmu.2021.780043

**Published:** 2021-12-21

**Authors:** Michelle Broekhuizen, Emilie Hitzerd, Thierry P. P. van den Bosch, Jasper Dumas, Robert M. Verdijk, Bas B. van Rijn, A. H. Jan Danser, Casper H. J. van Eijck, Irwin K. M. Reiss, Dana A. M. Mustafa

**Affiliations:** ^1^ Division of Neonatology, Department of Pediatrics, Erasmus University Medical Center, Rotterdam, Netherlands; ^2^ Division of Pharmacology and Vascular Medicine, Department of Internal Medicine, Erasmus University Medical Center, Rotterdam, Netherlands; ^3^ Division of Experimental Cardiology, Department of Cardiology, Erasmus University Medical Center, Rotterdam, Netherlands; ^4^ Department of Pathology, Erasmus University Medical Center, Rotterdam, Netherlands; ^5^ The Tumor Immuno-Pathology (TIP) Laboratory, Erasmus University Medical Center, Rotterdam, Netherlands; ^6^ Department of Obstetrics and Gynecology, Erasmus University Medical Center, Rotterdam, Netherlands; ^7^ Department of Surgery, Erasmus University Medical Center, Rotterdam, Netherlands

**Keywords:** placenta, preeclampsia, immune system, toll like receptor, complement, macrophage, mast cell

## Abstract

Preeclampsia is a severe placenta-related pregnancy disorder that is generally divided into two subtypes named early-onset preeclampsia (onset <34 weeks of gestation), and late-onset preeclampsia (onset ≥34 weeks of gestation), with distinct pathophysiological origins. Both forms of preeclampsia have been associated with maternal systemic inflammation. However, alterations in the placental immune system have been less well characterized. Here, we studied immunological alterations in early- and late-onset preeclampsia placentas using a targeted expression profile approach. RNA was extracted from snap-frozen placenta samples (healthy n=13, early-onset preeclampsia n=13, and late-onset preeclampsia n=6). The expression of 730 immune-related genes from the Pan Cancer Immune Profiling Panel was measured, and the data were analyzed in the advanced analysis module of nSolver software (NanoString Technology). The results showed that early-onset preeclampsia placentas displayed reduced expression of complement, and toll-like receptor (TLR) associated genes, specifically TLR1 and TLR4. Mast cells and M2 macrophages were also decreased in early-onset preeclampsia compared to healthy placentas. The findings were confirmed by an immunohistochemistry approach using 20 healthy, 19 early-onset preeclampsia, and 10 late-onset preeclampsia placentas. We conclude that the placental innate immune system is altered in early-onset preeclampsia compared to uncomplicated pregnancies. The absence of these alterations in late-onset preeclampsia placentas indicates dissimilar immunological profiles. The study revealed distinct pathophysiological processes in early-onset and late-onset preeclampsia placentas and imply that a tailored treatment to each subtype is desirable.

## 1 Introduction

Placentation and subsequent development of the placenta are essential processes that determine an uneventful pregnancy and good fetal outcome. Problems of placental development and adaptation can result in impaired fetal growth and preeclampsia affecting 2-8% of all pregnancies (1). The serious complications of preeclampsia can ultimately progress into maternal and fetal death, but if prevented, can also have other short-term and long-term negative impacts on the health of both mother and infant (2, 3). Apart from symptom relief and the undesirable option to terminate pregnancy and deliver the placenta, often necessitating preterm delivery of the child, preeclampsia cannot be treated successfully yet.

Although the etiology is incompletely understood, preeclampsia is generally considered a condition of placental insufficiency, accompanied by an exaggeration of the normal pregnancy-induced maternal systemic inflammatory response (4). The immune system plays important roles in placental development; directing trophoblast invasion, maintaining feto-maternal tolerance, and fighting invading pathogens when necessary (5). In a healthy pregnancy, immune cells are present in and around the placenta throughout all trimesters of pregnancy and are involved in interactions between the mother and the placenta. The placental bed, the decidua, is mainly occupied by cells of the innate immune system, including uterine natural killer (NK) cells, macrophages, mast cells, and dendritic cells (DCs) (5, 6). Adaptive immune cells including regulatory T cells and B cells are present in lower numbers and may be involved in maintaining a stable and appropriate immune response during later stages of pregnancy (5, 7, 8). The placenta itself mostly contains fetal innate immune cells, particularly fetal macrophages named Hofbauer cells. Immunological perturbations might be related to placental insufficiency, and multiple studies have shown elevated circulating inflammatory markers in pregnant women with preeclampsia (9–13). Preeclamptic placentas were also shown to express increased levels of the antiangiogenic factors endoglin and soluble fms-like tyrosine kinase-1 (14–16), which can both be shed into the maternal circulation. An increase in the concentrations of maternally circulating endoglin and sFlt1, and a subsequent decrease in placental growth factor (PlGF) signaling were identified as the major causal factors for the development of preeclampsia (1, 2). However, the involvement of the placental immune system is less clear as studies on placental immune cell alterations in preeclampsia have reported contradictory results, and the exact roles of placental immune cells in preeclampsia remain to be elucidated (5, 6).

Hence, in contrast to the well-defined maternal systemic inflammatory state in preeclampsia, placental immune alterations have been less well characterized. Tumors and placentas display striking similarities in developmental processes, and these similarities are increasingly recognized to be useful for understanding processes in either tissue (17, 18). A methodology that has emerged in the field of oncology uses the expression of immune-related genes to investigate the immune responses and the involved immune cell types and pathways in various tissue types (www.nanostring.com). A high sensitivity of this technique allows the identification of non-abundant immune cells, even in diseases with an immune-suppressive microenvironment like pancreatic cancers (19–21). Therefore, this technique provides a novel opportunity to investigate the immune profile of the placentas of women with preeclampsia.

While most studies cluster preeclampsia as one condition, it is generally acknowledged that two entities, with separate pathophysiological origins, can be distinguished: early-onset preeclampsia (onset <34 weeks of gestation), thought to originate from deficient placentation, and late-onset preeclampsia (onset ≥34 weeks of gestation), believed to be predominantly driven by maternal cardiovascular risk factors and placental insufficiency (2). Distinct placental mRNA expression profiles between early-onset preeclampsia and late-onset preeclampsia (22) suggest that inconsistent immunological findings in the placenta might be explained by a lacking subtype definition of preeclampsia. The identification of placental immunological differences may lead to the discovery of unidentified pathophysiological processes and potential targets for future therapeutic interventions. Therefore, with the present study, we aimed to reveal placental immune alterations between early-onset preeclampsia, late-onset preeclampsia, and healthy placentas. This study investigated this for the first time in the placenta by measuring an immune profiling gene expression array that was developed to investigate changes in cancer tissue, studying transcriptional, cellular and pathway alterations.

## 2 Methods

### 2.1 Study Cohort

All patients who were admitted to the Erasmus MC in Rotterdam gave written approval to participate in this study, prior to giving birth. The participants were either patients with singleton term pregnancies undergoing elective caesarean section or patients with preeclampsia, diagnosed based on the new ISSHP 2018 criteria, i.e., new-onset hypertension after 20 weeks of gestation (systolic blood pressure ≥140 mmHg or diastolic blood pressure ≥90 mmHg), accompanied by maternal organ dysfunction (e.g. proteinuria as defined by urinary protein/creatinine ratio >30 mg/mmol) and/or uteroplacental dysfunction defined as fetal growth restriction (23). Early-onset preeclampsia was defined based on the onset of symptoms <34 weeks of gestation, while late-onset preeclampsia ≥34 weeks of gestation. Clinical data were retrieved from the digital medical files. The study was exempted from medical approval according to the Dutch Medical Research with Human Subjects Law (MEC-2016-418 and MEC-2017-418). The gene expression analysis was performed in 16 healthy, 13 early-onset preeclampsia and 6 late-onset preeclampsia placentas. Since formalin-fixed, paraffin-embedded (FFPE) material was not available for all samples included in the gene expression analysis, an additional independent cohort of patients (9 healthy, 9 early-onset preeclampsia, and 6 late-onset preeclampsia), was added to validate the results by immunohistochemistry. Immunohistochemistry was carried out in a final cohort of 20 healthy, 20 early-onset preeclampsia and 10 late-onset preeclampsia patients.

### 2.2 Tissue Preparation

Within 20 minutes after delivery of the placenta, biopsies were taken from areas without obvious infarcts/necrosis, snap-frozen and stored at -80°C. In a limited number of samples from the same population, one additional full-thickness slice (1 cm in thickness) was collected per placenta and fixed in formalin, then embedded in paraffin (FFPE). Since FFPE samples were not available from all patients included in the gene expression profiling measurements, the number of FFPE samples was expanded as described in the previous section to confirm the results by immunohistochemistry.

### 2.3 RNA Extraction and Gene Expression Array Measurements

For RNA extraction, frozen biopsies were homogenized in RLT lysis buffer (Qiagen, Venlo, the Netherlands) with β-mercaptoethanol. After proteinase K (Invitrogen, Breda, the Netherlands) treatment for ten minutes at 55°C, RNA was extracted with the Qiagen RNA easy mini kit (Venlo, the Netherlands) according to the manufacturer’s instructions, eluted in RNA free water, and stored at -80°C. RNA Quality Control (QC) was measured using the 2100 Bioanalyzer (Agilent, CA, USA). NanoString Technology is based on detecting the genes of interest using 100 base pairs, but a cut-off value of 300 base pairs is necessary to increase the accuracy of measuring all the genes. Therefore, the total RNA concentration was corrected to include fragments seized between 300 and 4000 nucleotides. A total of 200 ng RNA was hybridized to the PanCancer Immune Profiling Panel for 17 hours at 67°C, following the manufacturing procedure (NanoString, Seattle, USA). The nCounter FLEX platform was used and genes were counted by scanning 490 Fields-of-view (FOV).

#### 2.3.1 Data Analysis

Data analysis was performed using the advanced analysis module (version 2.0) of nSolver™ software (version 4.0, NanoString Technology). The quality control of the measurements was done according to the general workflow used in the NanoString nCounter software analysis (24). Based on expression stability and minimum variance, thirty-three housekeeping genes (out of 40) were selected for normalization with the geNorm algorithm embedded in the advanced analysis module (**Supplementary Table S1**). The lower threshold of expression was calculated by multiplying the average expression of the negative controls by two. Genes that showed an expression count below the threshold in >80% of the samples were excluded from further analysis.

To identify differentially expressed genes in preeclampsia samples, healthy placenta samples were used as controls. Hence, data of the early-onset preeclampsia samples and that of the late-onset preeclampsia samples were compared to healthy control samples. In addition, to investigate the differences between the two groups of preeclampsia, data of late-onset preeclampsia were compared to that of early-onset preeclampsia. The normalized data were log_2_ transformed and the differentially expressed genes were identified using simplified negative binomial models, mixture negative binomial models, or log-linear models based on the convergence of each gene. The adjusted *P*-value was calculated using the Benjamini-Hoghberg method. Genes were considered differentially expressed when the adjusted *P*-value < 0.05 and the linear fold of change (FOC) > 1.5 in any direction.

#### 2.3.2 Immune Cell Subset Analysis

Specific and unique cell type marker genes were used to define multiple immune cell subtypes (**Supplementary Table S2**). The marker genes were selected based on the default settings of the advanced analysis module of nSolver, combined with placenta-specific immune cell genes identified in single-cell RNA sequencing studies (25–27). By using marker genes to define the cell types we assume that the immune cell’s genes are expressed in that specific cell type only (mutually exclusive) and that the expression of the marker genes is equal between cells for all samples. As a quality check of the cell definition based on previous research, pairwise similarities between the marker genes of each cell type were considered sufficient if R ≥ 0.6 (**Supplementary Figure S1**) (28). Unfortunately, dendritic cell marker genes did not pass the quality control criteria, and therefore this cell type was not analyzed. The cell type marker genes were used to calculate a cell type score for each sample.

#### 2.3.3 Pathway Analysis

Pathway alterations were analyzed by clustering genes into pathways using the default settings of the PanCancer Immune Profiling advanced analysis module in nSolver. The pathway scores were calculated for each sample as the square root of the average squared t-statistic of all genes in the corresponding pathway.

The database for annotation, visualization, and integrated discovery (DAVID) v6.8 (29–31) was used as an alternative method to functionally annotate and cluster genes. For this analysis, separate lists were used containing either the downregulated or upregulated genes with an unadjusted *P*-value < 0.05, comparing early-onset preeclampsia and late-onset preeclampsia to healthy placentas. Genes that were related to either TLR signaling, the complement pathway or chemotaxis-associated cytokines as identified in DAVID were used to perform unsupervised hierarchical heatmap clustering using Ward’s method and visualized using the ComplexHeatmap package in R (32).

### 2.4 Immunohistochemistry

#### 2.4.1 M1/M2 Macrophage Staining

Sections of 4µm of FFPE samples were used for immunohistochemistry analysis. To visualize M1 and M2 macrophage subsets in placental tissue, the chromogenic duplex staining was done with CD68 and CD163 by an automated staining procedure using the Ventana Benchmark Discovery (Ventana Medical Systems Inc.). In brief, following deparaffinization and heat-induced antigen retrieval with CC1 (#950-500, Ventana) for 40 minutes at 95°C, the tissue samples were firstly incubated with CD68 for 60 minutes at 37˚C, followed by detection with anti-mouse HQ (#760-4814, Ventana) for 16 minutes and subsequently anti-HQ HRP (#760-4820), and visualized with Discovery Purple (#760-229, Ventana) for 32 minutes. An antibody denature step was performed using CC2 (#950-123, Ventana) for 20 minutes at 100˚C. Secondly, CD163 was incubated for 32 minutes at 37˚C, followed by detection with anti-mouse HQ and subsequently anti-HQ HRP, and visualized with Discovery Teal (#760-247, Ventana) for 32 minutes. Finally, all slides were counterstained with hematoxylin II (#760-2208) and bluing reagents (#760-2037) for 4 minutes. Antibody information and clonality can be found in **Supplementary Table S3**.

#### 2.4.2 Mast Cell Staining

To visualize mast cells in placental tissue, sequential 4 µm thick (FFPE) sections were stained by conventional immunohistochemistry for tryptase, using the optiview universal DAB detection kit (#760-700, Ventana). In brief, following deparaffinization and heat-induced antigen retrieval with CC1 (#950-500, Ventana) for 64 minutes, the tissue samples were incubated with the antibody of interest (**Supplementary Table S3**) for 32 minutes at 37°C. Incubation was followed by hematoxylin II counterstain for 8 minutes and then a blue coloring reagent for 8 minutes according to the manufactures instructions (Ventana).

For analysis, the immunohistochemistry slides were scanned on a NanoZoomer 2.0-HT (Hamamatsu Photonics K.K.). In NDPview (version 2.5.19 Hamamatsu Photonics K.K.), 1 mm^2^ areas were selected in each slide, three in the central villus part of the placenta, and three covering the decidua. Only areas with open villus structures were selected, avoiding infarcted tissue and placental septa. The images were imported as separate JPEG images into ImageJ (version 1.53a Wayne Rasband, USA). The number of positive cells with visible nuclei was counted by hand. In the central areas of the placenta, only cells inside the villi were counted, excluding cells in the intervillous space. To normalize for variation in decidual thickness, the decidual area was drawn by hand, measured, and cell counts were reported as cells/mm^2^. For the combined CD68/CD163 staining, cells were counted as either double-positive cells or CD68 single-positive cells. These cells were predefined as M2, and M1 macrophages, respectively. All analyses were performed blinded by one researcher. Power analysis for immunohistochemistry revealed a requirement of n=20 per group to obtain statistical significance at α=0.05 and β=0.2. However, there was not enough late-onset preeclampsia material available, and one early-onset preeclampsia placenta had too many infarcts and was therefore excluded.

### 2.5 Statistical Tests

Unless stated otherwise, analyses were conducted and images were produced in Graphpad Prism 8 or R 4.1 using the ggplot2 package (33, 34). The cell type scores, pathway scores, and immunohistochemistry results were statistically tested between groups using a Kruskall-Wallis test with Tukey’s multiple comparisons test. A result was considered statistically significant if the *P*-value < 0.05.

## 3 Results

### 3.1 Clinical Characteristics

Out of the 36 mRNA samples which were measured by the gene expression array, four samples had to be excluded from further analysis. One sample was retrospectively excluded due to misclassification as late-onset preeclampsia while the patient suffered from pregnancy-induced hypertension. Three healthy control samples were excluded because these did not meet the quality control criteria after gene expression measurement. The binding density in these samples, reflecting the total percentage of detected genes, was <0.2 compared to >0.7 in the 32 included samples, this was also reflected by significantly lower counts of positive controls and housekeeping genes (**Supplementary Figure S2**). The remaining study population included samples of patients with early-onset preeclampsia (n=13), late-onset preeclampsia (n=6), and uncomplicated pregnancies (n=13) (**Table 1**).

**Table 1:** Clinical characteristics of the healthy, early-onset preeclampsia (eoPE) and late-onset preeclampsia (loPE) patients included in the gene expression analysis and immunohistochemistry studies.

Gene expression analysis	Healthy (n = 13)	eoPE (n = 13)	loPE (n = 6)
Maternal age (years)	35 (29 - 39)	30 (26 - 33)	32 (30 - 38)
Parity (n)			
0	0	10*	3^†^
1	6	3	2
>1	7	0	1
Ethnicity (n Caucasian/other)	8/5	8/5	3/3
Body mass index (kg/m^2^)	24.7 (22.5 - 33.4)	25.2 (22.2 - 32.2)	29.4 (21.5 - 32.4)
Diastolic blood pressure (mmHg)	80 (70 - 80)	109 (102 - 114)*	100 (99 - 106)^†^
Systolic blood pressure (mmHg)	121 (113 - 135)	170 (142 - 180)*	150 (136 - 164)
Mean arterial pressure (mmHg)	95 (85 - 99)	129 (115 - 140)*	120 (111 - 124)^†^
Urinary protein/creatinine ratio (mmol/mg)	not measured	297 (155 - 768)	109 (102 - 114)^‡^
Mode of delivery (n caesarean/spontaneous)	13/0	12/1	6/0
Gestational age (weeks)	39.0 (38.6 - 39.1)	30.6 (28.6 - 31.8)*	36.0 (35.1 - 37.0)
First moment of PE diagnosis (weeks)	not applicable	29.4 (27.1 - 30.7)	34.7 (34.3 - 35.3)^‡^
Sex (n female/male)	6/7	6/7	2/4
Fetal birth weight (g)	3515 (2963 - 3940)	1111 (925 - 1380)*	2333 (1851 - 3956)^‡^
Fetal birth centile (n)			
<3^rd^	0	5*	1
3^rd^ -10^th^	1	3	2
≥10^th^	12	5	3
Placenta weight (g)	708 (628 - 783)	286 (243 - 343)*	428 (351 - 686)
**Immunohistochemistry**	**Healthy (n = 20)**	**eoPE (n = 19)**	**loPE (n = 10)**
Maternal age (years)	35 (30 - 38)	30 (26 - 33)*	31 (29 - 36)
Parity (n)			
0	2	12*	2
1	11	7	6
>1	7	0	2
Ethnicity (n Caucasian/other)	11/9	12/7	4/6
Body mass index (kg/m^2^)	24.7 (22.8 - 32.1)	24.6 (22.2 - 31.2)	27.2 (24.8 - 31.6)
Diastolic blood pressure (mmHg)	80 (70 - 80)	105 (100 - 115)*	103 (99 - 110)^†^
Systolic blood pressure (mmHg)	123 (110 - 130)	160 (142 - 173)*	150 (138 - 160)^†^
Mean arterial pressure (mmHg)	93 (85 - 97)	123 (117 - 135)*	115 (109 - 123)^†^
Urinary protein/creatinine ratio (mmol/mg)	not measured	124 (34 - 580)	96 (39 - 148)
Mode of delivery (n caesarean/spontaneous)	20/0	18/1	8/2
Gestational age (weeks)	39.0 (38.7 - 39.1)	29.8 (28.4 - 31.6)*	36.6 (36.3 - 37.5)^†^‡
First moment of PE diagnosis (weeks)	not applicable	28.4 (26.7 - 30.6)	35.6 (35.1 - 36.9)^‡^
Sex (n female/male)	8/12	13/6	4/6
Fetal birth weight (g)	3535 (3288 - 3870)	1080 (884 - 1430)*	2773 (2210 - 3480)^‡^
Fetal birth centile (n)			
<3^rd^	0	7*	0 †
3^rd^ -10^th^	0	6	4
≥10^th^	20	6	6
Placenta weight (g)	649 (491 - 772)	280 (228 - 337)*	512 (326 - 676)^‡^

Data are shown as n (number of cases) or median (interquartile range). *P < 0.05 eoPE vs healthy, ^†^P < 0.05 loPE vs healthy, ^‡^P < 0.05 loPE vs eoPE as determined with an independent Kruskal-Wallis test with Bonferroni post hoc test or with a Chi square test.

### 3.2 More Immune-Related Gene Expression Alterations in Early-Onset, Than in Late-Onset Preeclampsia Placentas

**Figure 1:**
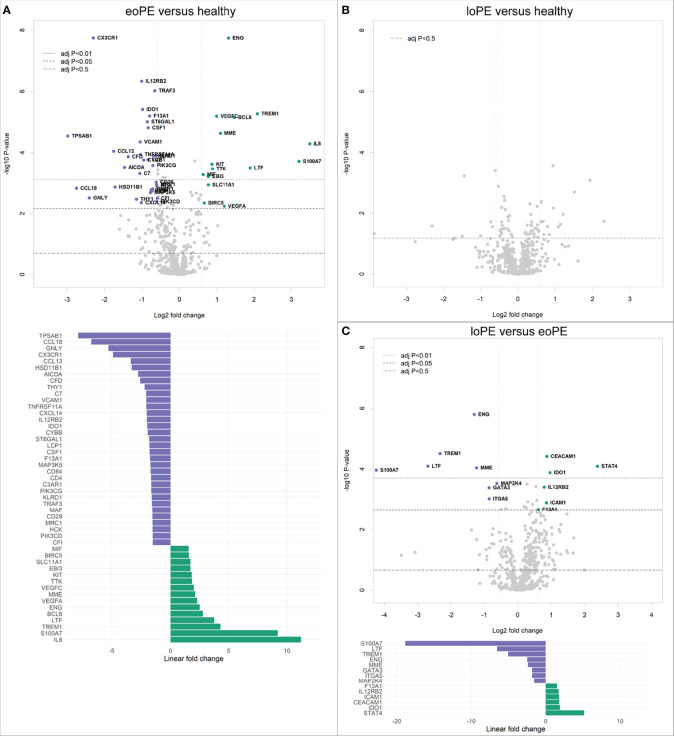
Gene expression is altered in early-onset preeclampsia (eoPE) compared to healthy and late-onset preeclampsia (loPE) placentas. Differential gene expression analyses in early-onset preeclampsia compared to healthy **(A)**, late-onset preeclampsia compared to healthy **(B)**, and late-onset compared to early-onset preeclampsia placentas **(C)**. Points with annotation in the volcano plots depict genes with an adjusted P-value < 0.05 and FOC > 1.5. The bottom graphs in panels **(A, C)** display the linear fold changes of the differentially expressed genes. The annotations represent the official gene names.

Analysis of 730 individual genes revealed 33 downregulated, and 15 upregulated genes in early-onset preeclampsia compared to healthy controls (**Figure 1A**). Late-onset preeclampsia placentas did not display any immune-related gene expression differences compared to healthy controls after correction for multiple testing (**Figure 1B**). However, eight downregulated and six upregulated genes were found in late-onset compared to early-onset preeclampsia placentas (**Figure 1C**).

### 3.3 Decreased Toll-Like Receptor (TLR) and Complement Signaling in Early-Onset Preeclampsia Placentas

**Figure 2:**
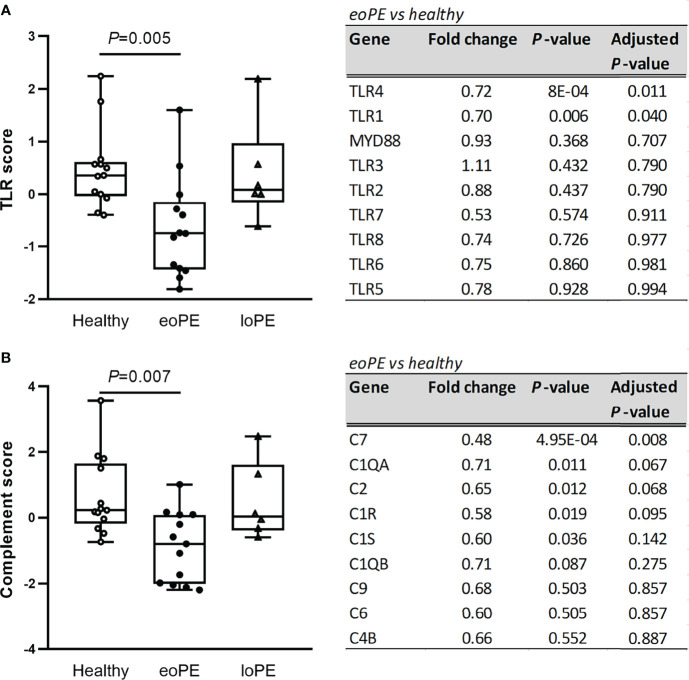
TLR- and complement-associated gene expression are diminished in early-onset preeclampsia (eoPE) placentas, but not in late-onset preeclampsia (loPE). The TLR score **(A)** and complement score **(B)** were calculated in each group using the genes displayed in the adjacent tables. Statistical significance was determined using the Kruskal-Wallis test followed by Tukey’s multiple comparisons tests.

Pathway analysis of the gene expression data, using multiple predefined pathways (**Supplementary Table S4**), revealed decreased expression of TLR-associated genes in early-onset preeclampsia compared to healthy placentas (*P*=0.005), mainly due to reduced expression of TLR4 and TLR1 (**Figure 2A**). Additionally, complement-associated genes were reduced in early-onset preeclampsia compared to healthy placentas (*P*=0.007, **Figure 2B**), including complement 7 (C7), complement C1q A chain (C1QA), complement 2 (C2), and complement C1r (C1R). None of the predefined pathways were statistically significantly altered in late-onset preeclampsia.

**Figure 3:**
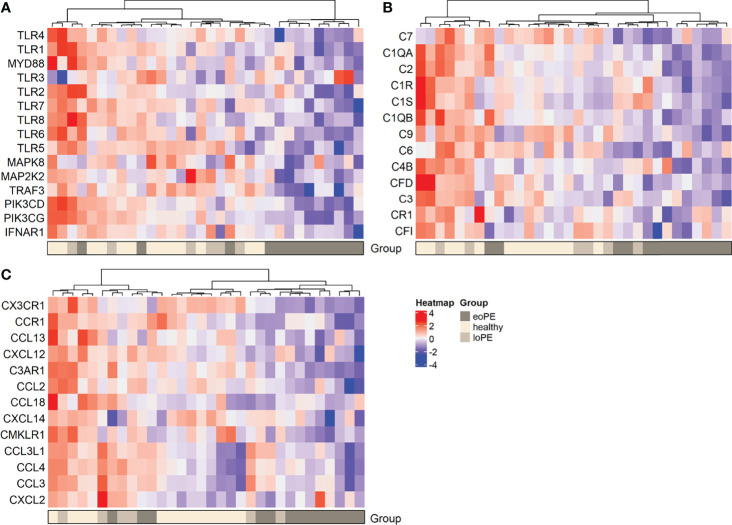
Heatmap hierarchical clustering displays lower expression of genes associated with the TLR pathway **(A)**, complement activation **(B)**, and chemokines **(C)** in early-onset preeclampsia placentas. Red depicts increased and blue depicts decreased mRNA expression scaled to the gene average. eoPE, early-onset preeclampsia; loPE, late-onset preeclampsia.

DAVID functional clustering and pathway analysis was performed using the genes with an unadjusted *P*-value < 0.05 to investigate further interactions. In agreement with the gene expression findings, TLR signaling and complement activation were found to be reduced in early-onset preeclampsia compared to healthy placentas (**Supplementary Table S5**). In addition, “responses against non-self tissue” and “chemotaxis-associated chemokines” were identified as downregulated pathways in early-onset preeclampsia placentas. Genes that were upregulated in early-onset preeclampsia did not result in any significant pathway interactions. The pathways “inflammatory signaling” and “increased chemotaxis” networks were found to be significantly upregulated in late-onset preeclampsia compared to healthy placentas (**Supplementary Table S5**). No significant pathway interactions were obtained concerning the downregulated genes in late-onset preeclampsia. To highlight the genes which were involved in the most significant pathways, hierarchical heatmap clustering was performed and shown in **Figure 3**.

### 3.4 Reduced Innate Immune Cell Populations in Placentas From Early-Onset Preeclampsia Patients

**Figure 4:**
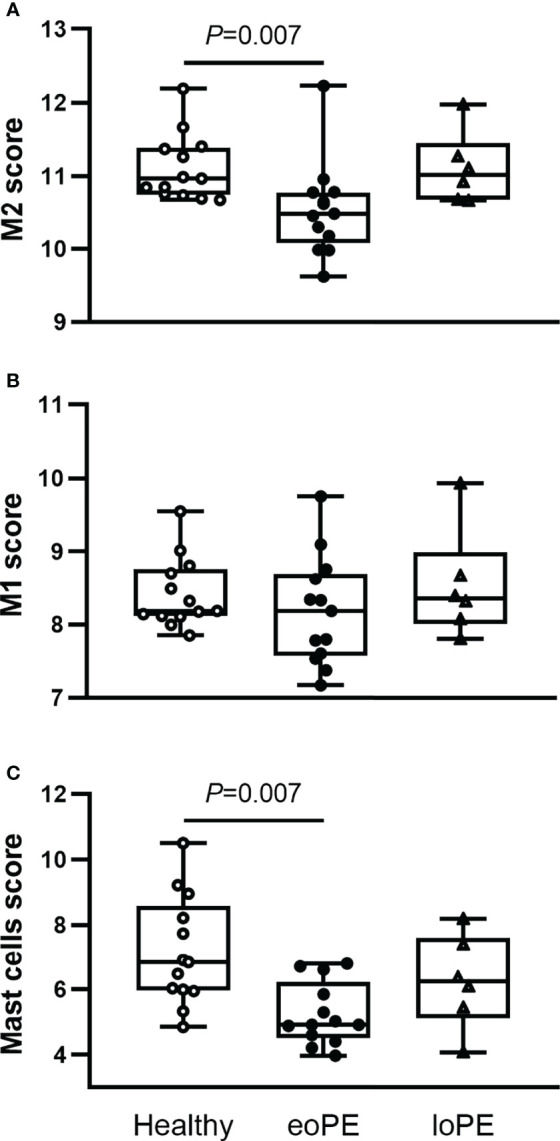
Mast cells and M2 macrophages are less abundant in early-onset preeclampsia (eoPE) placentas. Cell type scores were calculated based on the gene expression of CD163, MRC1, C1QA and C1QB for M2 macrophages **(A)**, CD86 and MSR1 for M1 macrophages **(B)**, TPSAB1 for mast cells **(C)**. The lines represent median and interquartile ranges. Statistical significance was determined using the Kruskal-Wallis test followed by Tukey’s multiple comparisons tests. eoPE, early-onset preeclampsia; loPE, late-onset preeclampsia.

Using the gene expression analysis, we identified multiple immune cell subtypes in the placenta (**Supplementary Table S2**). The genes that define NK CD56dim, regulatory T cell, Th1 cell, and CD8 T cell signals were expressed below the threshold of expression and therefore not identified nor compared between the samples. M2 macrophages (CD136^+^MRC1^+^C1QA^+^C1QB^+^, *P*=0.007) and mast cells (TPSAB1, *P*=0.007) were decreased in early-onset preeclampsia samples, while M1 macrophages (CD86^+^MSR1^+^) were not altered based on gene expression (**Figures 4A–C**). Although the expression of the B cell marker gene CD19 was increased in late-onset preeclampsia versus early-onset preeclampsia (*P*=0.015), it did not differ in either condition when compared to healthy placentas. All other investigated cell types did not statistically significantly differ between our groups.

**Figure 5:**
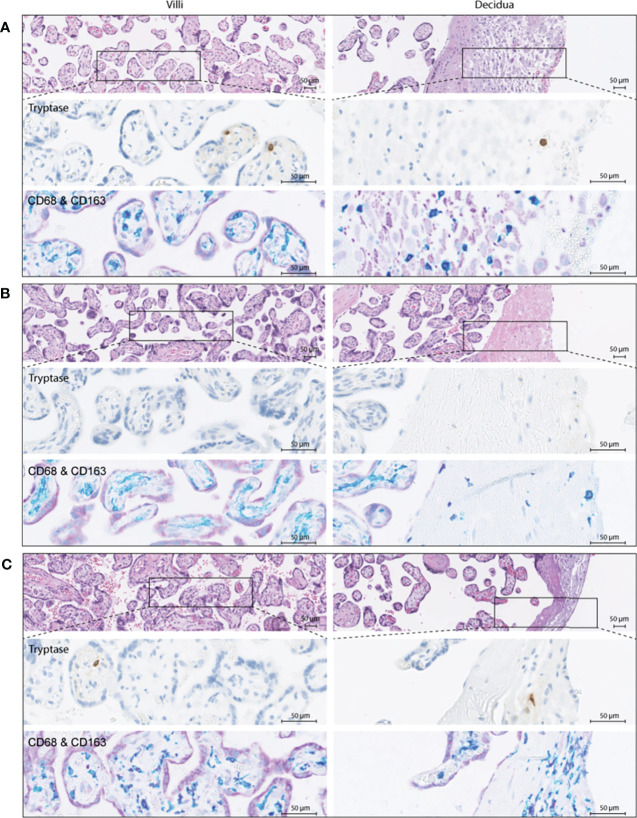
Immunohistochemistry examples of healthy **(A)**, early-onset preeclampsia (eoPE, **B**) and late-onset preeclampsia (loPE, **C**) placentas. In each panel the top images were stained for hematoxylin and eosin, the middle images for tryptase to visualize mast cells, and the bottom images for CD68 (teal) & CD163 (purple) to visualize macrophages. CD68^+^CD163^+^ (dark purple) cells were defined as M2 macrophages, CD68^+^CD163^-^ (teal) cells as M1 macrophages. The left panels display the placental villi, and the right panels display the decidua.

**Figure 6:**
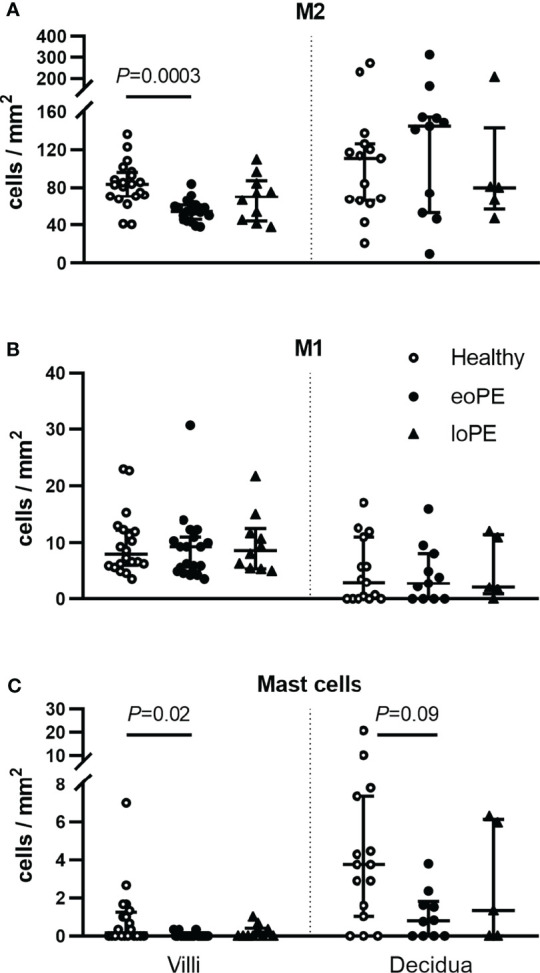
Mast cells and M2 macrophages are less abundant in the villi of early-onset preeclampsia (eoPE) placentas. M2 macrophages (CD68^+^CD163^+^, **A**), M1 macrophages (CD68^+^CD163^-^, **B**) and mast cells (tryptase, **C**) were stained by immunohistochemistry and quantified in the villi and in the decidual area. The decidua was missing in 5 healthy, 9 early-onset preeclampsia and 5 late-onset preeclampsia slides. The lines represent median and interquartile ranges. Statistical significance was determined using the Kruskal-Wallis test followed by Tukey’s multiple comparisons tests. eoPE, early-onset preeclampsia; loPE, late-onset preeclampsia.

To verify these findings, immunohistochemistry was performed in a partially independent cohort as described in the Methods and defined in **Table 1**. Examples of the M1/M2 and mast cell immunohistochemistry images are shown in **Figure 5**, and the quantification of the immunohistochemistry is displayed in **Figure 6**. M2 macrophages were more abundant inside the villi of healthy compared to early-onset preeclampsia placentas (*P*=0.0003, **Figure 6A**), but not different in the decidua. M1 macrophages were not altered between the various groups (**Figure 6B**). While a few mast cells were detected inside the villi of the healthy placentas, they were rarely detected in the villi of early-onset preeclampsia placentas (*P*=0.02, **Figure 6C**). These early-onset preeclampsia placentas displayed a trend towards reduced mast cell numbers in the decidua as well (*P*=0.09, **Figure 6C**).

## 4 Discussion

Placental immunological processes are important for the development of the placenta and fetus, and the maintenance of a tolerogenic environment. Disturbances in these processes could lead or contribute to preeclampsia, a condition that has been associated with a maternal systemic inflammatory state. However, placental immune alterations have been less well characterized. Data from the present study suggest that in contrast to the maternal systemic inflammatory state, placental innate immune factors are compromised in early-onset preeclampsia. Absence of these placental immune alterations in late-onset preeclampsia, suggests that both subtypes have dissimilar placental immune profiles, confirming distinct pathophysiological processes. It has been shown that placental dysfunction contributes to the maternal (inflammatory) syndrome in both subtypes. Nevertheless, the results of this study indicate that a maternal systemic inflammatory state does not necessarily translate to an inflammatory placenta.

Findings from the current study agree with the distinct gene expression profiles that have been identified between placentas from women with early-onset preeclampsia and late-onset preeclampsia (22, 35), and further extends this knowledge by identifying specific immune alterations. The placentas from patients with early-onset preeclampsia displayed increased placental endoglin expression as shown by the differential gene expression analysis, and in agreement with previous studies in women with severe preeclampsia (15, 16). However, earlier studies have also shown that placental expression of endoglin was increased in early-onset preeclampsia versus late-onset preeclampsia, and that elevated levels of circulating endoglin were more pronounced in preterm versus term preeclampsia (36, 37). Therefore, endoglin seems to have a more significant role in early-onset preeclampsia than in late-onset preeclampsia. Another gene that was differentially expressed between early-onset preeclampsia and late-onset preeclampsia was indoleamine 2,3-dioxygenase (IDO)1. The decreased IDO1 expression in early-onset preeclampsia is in agreement with previous studies (38–41), and based on the current study, seems to be specific to early-onset preeclampsia. IDO1 is the first enzyme of the kynurenine pathway of which we recently reviewed its placental functions and alterations in preeclampsia (42). The kynurenine pathway provides an important source of *de novo* NAD^+^ synthesis, and is involved in vascular development and functioning, anti- and pro-oxidative processes, and immune regulation. However, the function of the kynurenine pathway is mainly determined by tryptophan transporters (38), and by the spatial localization of IDO1 (42). The spatial distribution was not preserved in this study, therefore, it impossible to draw any further conclusions on the differential roles of IDO1 in preeclampsia.

Early-onset preeclampsia placentas displayed a decreased expression of TLR4, in agreement with earlier research (43), while studies in mild preeclampsia have reported either increased or unaltered TLR4 expression (44–49), similar to our late-onset preeclampsia patients. A role for reduced TLR4 signaling in early-onset preeclampsia was also suggested by a higher prevalence of allelic TLR4 gene variants with attenuated function in women with a history of early-onset preeclampsia (50). TLR4 can recognize several other pathogen-associated molecular patterns, in addition to its classical anti-microbial function (51, 52). The absence of visible infections, and the expression of TLR4 in many placental cells including trophoblasts (data not shown), suggests that TLR4 has other roles in the placenta than microbial defense only. In contrast to a decreased placental TLR4 expression as identified in the present study, previous studies have shown that the expression of TLR4 was elevated in the circulating innate immune cells of women with preeclampsia (53, 54). This suggests that the regulation of TLR4 is differently altered in preeclampsia in the fetal compared to the maternal compartment.

The present study confirmed that C7 is a potential gene candidate to discriminate early-onset from late-onset preeclampsia (22), and suggests a generally compromised complement system in early-onset preeclampsia placentas. Downregulation of C7 in the placentas of women with preeclampsia, but not in pregnancies with fetal growth restriction only, suggests this is specific to preeclampsia (55). While under normal conditions the complement system plays a role in regulating tolerance, apoptosis, angiogenesis, inflammation and clearance of free fetal DNA (56), locally decreased C1q production leads to decreased trophoblast invasion (57). Moreover, pregnant C1q deficient mice display a preeclampsia-like phenotype (58). Hence, insufficient production of complement factors can lead to impaired placental development and might precede early-onset preeclampsia.

A lower abundance of mast cells in the villi of early-onset preeclampsia placentas is in agreement with previous research (59), and seems specific to early-onset preeclampsia (60, 61). Mast cells are a source of angiogenic factors (62), and their depletion results in aberrant spiral artery remodeling and eventually fetal growth restriction (63). An increased mast cell number is related to a higher microvascular density in diabetic placentas (64), which was also associated with increased CX3CR1 expression (65). CX3CR1, the most statistically significant downregulated gene in the present study, is a transmembrane protein on immune cells, involved in cell adhesion, migration, and angiogenesis through interaction with its ligand CX3CL1 (fractalkine), and activation of hypoxia-inducible factor 1α (HIF1α) and vascular endothelial growth factor (VEGF) signaling (66). Hence, reductions in mast cells and CX3CR1 expression might contribute to impaired villous vascular development in early-onset preeclampsia placentas. However, CX3CR1 and the mast cell marker TPSAB1 did not correlate in the current gene expression analysis (data not shown), suggesting that CX3CR1 is expressed by other placental (immune) cells as well.

This study presents for the first time a decrease of M2 macrophages, presumably Hofbauer cells, in the villi of early-onset preeclampsia placentas. Reduced M2 macrophage numbers have been reported in fetal growth restriction and pregnancy-induced hypertension (67, 68). Since this already occurred in the first trimester (68), M2 alterations seemed to precede pathological placental development. While at the beginning of pregnancy M2 macrophages play a role in trophoblast invasion and spiral artery remodeling, decidual M2 macrophages were not altered in the current study. However, loss of M2 macrophages inside the villi, might be related to impaired clearance of apoptotic bodies, and insufficient protection of the fetus against intrauterine infection (69, 70). Earlier studies have shown that total macrophage numbers were not altered in preeclampsia (presumably late-onset based on the gestational age) and fetal growth restriction, but increased in the case of a decreased fetal growth rate (71, 72), suggesting that the lower M2 numbers in early-onset preeclampsia are not related to the co-occurrence of fetal growth restriction. Additionally, T cell infiltration was increased in preeclampsia and fetal growth restriction (71), although CD8 T cell genes were expressed below the detection limit in our study.

Recently, pyroptosis was identified as one of the key events that can contribute to systemic sterile inflammation specifically in patients with early-onset preeclampsia (73). Pyroptosis can trigger inflammatory responses independent of TLR4 and can cause membrane rupture and consequent release of pro-inflammatory cytokines like IL-1β, IL-18, and IL-33 (73, 74). Pyroptosis has been documented mainly in cells of the myeloid lineage such as macrophages, but recent work has demonstrated that pyroptosis occurs in primary human trophoblasts in the placenta from women with early-onset preeclampsia (75). Since pyroptosis was not investigated in the present study, its link to the currently identified alterations remains subject for future studies.

The current study has a few limitations. Firstly, an influence of the lower gestational age in women with early-onset preeclampsia cannot be ruled out. However, concurrent results between the present study and data from Nevalainen et al. (22) and Than et al. (35), who did use gestational age-matched controls, and absence of a correlation between gestational age and our most important findings within the early-onset preeclampsia group (data not shown), argue the results are unrelated to pregnancy duration. Secondly, as part of clinical care to improve fetal long maturation, twelve out of the thirteen early-onset preeclampsia patients in the gene expression analysis cohort received one or two dosages of the corticosteroid betamethasone within 48 hours of each other. Additional analyses reassured that the results were not confounded by betamethasone (**Supplementary Figure S3**). Thirdly, it should be acknowledged that the lower number of late-onset preeclampsia samples may have resulted in power loss for this group, and therefore potentially contributed to the absence of alterations. For example, the immunohistochemistry results revealed large variations in M2 macrophage and mast cell numbers in both late-onset preeclampsia and healthy samples, but the power to detect a statistically significant difference compared to early-onset preeclampsia was probably not achieved in late-onset preeclampsia, because of the lower number of included samples. Lastly, in the current study mRNA from whole placental tissue was used that included fetal and maternal blood, and some decidual tissue. This makes it impossible to discern which tissue compartments contributed to the identified differences. Moreover, rather than causal, certain immune components might have been downregulated in response to a maternal systemic inflammatory condition, although this generally described pro-inflammatory state may not be preeclampsia-specific but confounded by an increased maternal body mass index (76), which did not differ between our groups. Studying the spatial alterations of the placental immune compartments in both early-onset preeclampsia and late-onset preeclampsia will provide substantial information to understand the disease. In addition, changes in the placental microenvironment should be connected to those in the circulation, especially in early-onset preeclampsia, to investigate whether those women truly experience systemic inflammation.

In conclusion, the present study identified alterations in the innate immune system in placentas of women with early-onset preeclampsia, reflected by downregulated TLR and complement pathways, and a lower abundance of mast cells and M2 macrophages in the fetal villi. The identification of placental immune alterations in early-onset preeclampsia but not in late-onset preeclampsia asserts distinct pathophysiological processes, underlining the need to investigate them separately. These data provide a basis to direct future research into the placental innate immune system, and to discriminate between early-onset and late-onset preeclampsia, to eventually interfere and tailor treatment to these distinct pathological pregnancy conditions.

## Data Availability Statement

The data presented in the study are deposited in the Gene Expression Omnibus (GEO) repository, accession number GSE190639, https://www.ncbi.nlm.nih.gov/geo/query/acc.cgi?acc=GSE190639.

## Ethics Statement

The study was reviewed by the Medische Ethische Toetsings Commisie Erasmus MC and exempted from medical approval according to the Dutch Medical Research with Human Subjects Law (MEC-2016-418 and MEC-2017-418). The participants provided their written informed consent to participate in this study.

## Author Contributions

The study was designed by MB, IR, and DM. Data collection was performed by MB, EH, TB, and JD, whilst data analysis was done by MB and DM. MB, EH, RV, BR, and CE contributed to the data interpretation. MB wrote the manuscript and designed the figures. All authors contributed to the article and approved the submitted version.

## Funding

This work was partially supported by the Support Casper Foundation (www.supportcasper.org).

## Conflict of Interest

The authors declare that the research was conducted in the absence of any commercial or financial relationships that could be construed as a potential conflict of interest.

## Publisher’s Note

All claims expressed in this article are solely those of the authors and do not necessarily represent those of their affiliated organizations, or those of the publisher, the editors and the reviewers. Any product that may be evaluated in this article, or claim that may be made by its manufacturer, is not guaranteed or endorsed by the publisher.
