# Supplementary material for: The Placental Innate Immune System Is Altered in Early-Onset Preeclampsia, but Not in Late-Onset Preeclampsia
Source: Front Immunol. 2021 Dec 21;12:780043. doi: 10.3389/fimmu.2021.780043 (PMC8724430; doi:10.3389/fimmu.2021.780043)

Supplementary Material

**Table S1. Housekeeping genes which were used for normalization of the gene expression data.**

| **Housekeeping genes** | **Gene** |
| --- | --- |
| FCF1 | NM_015962.4:1022 |
| EDC3 | NM_001142443.1:925 |
| MRPS5 | NM_031902.3:390 |
| TLK2 | NM_006852.2:2335 |
| PRPF38A | NM_032864.3:335 |
| DDX50 | NM_024045.1:1185 |
| TBP | NM_001172085.1:587 |
| DHX16 | NM_001164239.1:2490 |
| EIF2B4 | NM_172195.3:1390 |
| MTMR14 | NM_022485.3:720 |
| ERCC3 | NM_000122.1:1950 |
| CNOT10 | NM_001256741.1:1962 |
| CNOT4 | NM_001190848.1:795 |
| TMUB2 | NM_024107.2:1485 |
| AMMECR1L | NM_001199140.1:3564 |
| ZC3H14 | NM_001160103.1:2690 |
| SF3A3 | NM_006802.2:2060 |
| ZKSCAN5 | NM_014569.3:3688 |
| TRIM39 | NM_021253.3:3140 |
| SAP130 | NM_024545.3:3090 |
| AGK | NM_018238.3:816 |
| COG7 | NM_153603.3:1492 |
| SDHA | NM_004168.1:230 |
| POLR2A | NM_000937.2:3775 |
| ZNF143 | NM_003442.5:925 |
| USP39 | NM_001256725.1:806 |
| NOL7 | NM_016167.3:335 |
| ABCF1 | NM_001090.2:850 |
| HDAC3 | NM_003883.2:1455 |
| HPRT1 | NM_000194.1:240 |
| TUBB | NM_178014.2:320 |
| GUSB | NM_000181.1:1350 |
| PPIA | NM_021130.2:925 |

**Table S2. The markers used for cell type definition in the advanced analysis module in nSolver.**

| **Cell type** | **Marker(s)** |
| --- | --- |
| Leukocytes | CD45 |
| B cells | CD19 |
| T cells | CD3D |
| Exhausted CD8 | LAG3 |
| Mast cells | TPSAB1 |
| Neutrophils | CSF3R, S100A12 |
| Macrophages | CD68, CD84 |
| M1 | CD86, MSR1 |
| M2 / Hofbauer cells | CD163, MRC1, C1QA, C1QB |
| NK cells | XCL2, KLRB1, KLRC1, GZMB, GZMA |

Natural killer (NK) CD56dim, regulatory T cells, Th1 cells, and CD8 T cells were discarded due to signals below the lower threshold of expression. Data on dendritic cell marker genes did not pass the quality control criteria (R≥0.6), and therefore this cell type was not analyzed.

**Table S3. Antibody information and clonality.**

| **Antibody** | **Type** | **Concentration** | **Company** | **Clone** |  |  |
| --- | --- | --- | --- | --- | --- | --- |
| CD68 | Anti-mouse | 0.4 µg/ml | Ventana | KP1 |  |  |
| CD163 | Anti-mouse | 0.2 µg/ml | Ventana | MRQ-26 |  |  |
| Tryptase | Anti-mouse | 0.05 µg/ml | Cell Marque | G3 |  |  |

**Table S4. Pathways investigated using the PanCancer immune profiling advanced analysis module in nSolver.**

| **Pathways** |  |
| --- | --- |
| Adhesion | Leukocyte Functions |
| Antigen Processing | Macrophage Functions |
| B-Cell Functions | Microglial Functions |
| Cell Cycle | NK Cell Functions |
| Cell Functions | Pathogen Defense |
| Chemokines | Regulation |
| Complement | Senescence |
| CT Antigen | T-Cell Functions |
| Cytokines | TLR |
| Cytotoxicity | TNF Superfamily |
| Interleukins | Transporter Functions |

**Table S5. Summary of the most important DAVID pathway analysis findings.**

**Figure S1.** **Correlations and slopes of the markers used to define macrophages, M1 macrophages, M2 macrophages, and natural killer (NK) cells.**

**
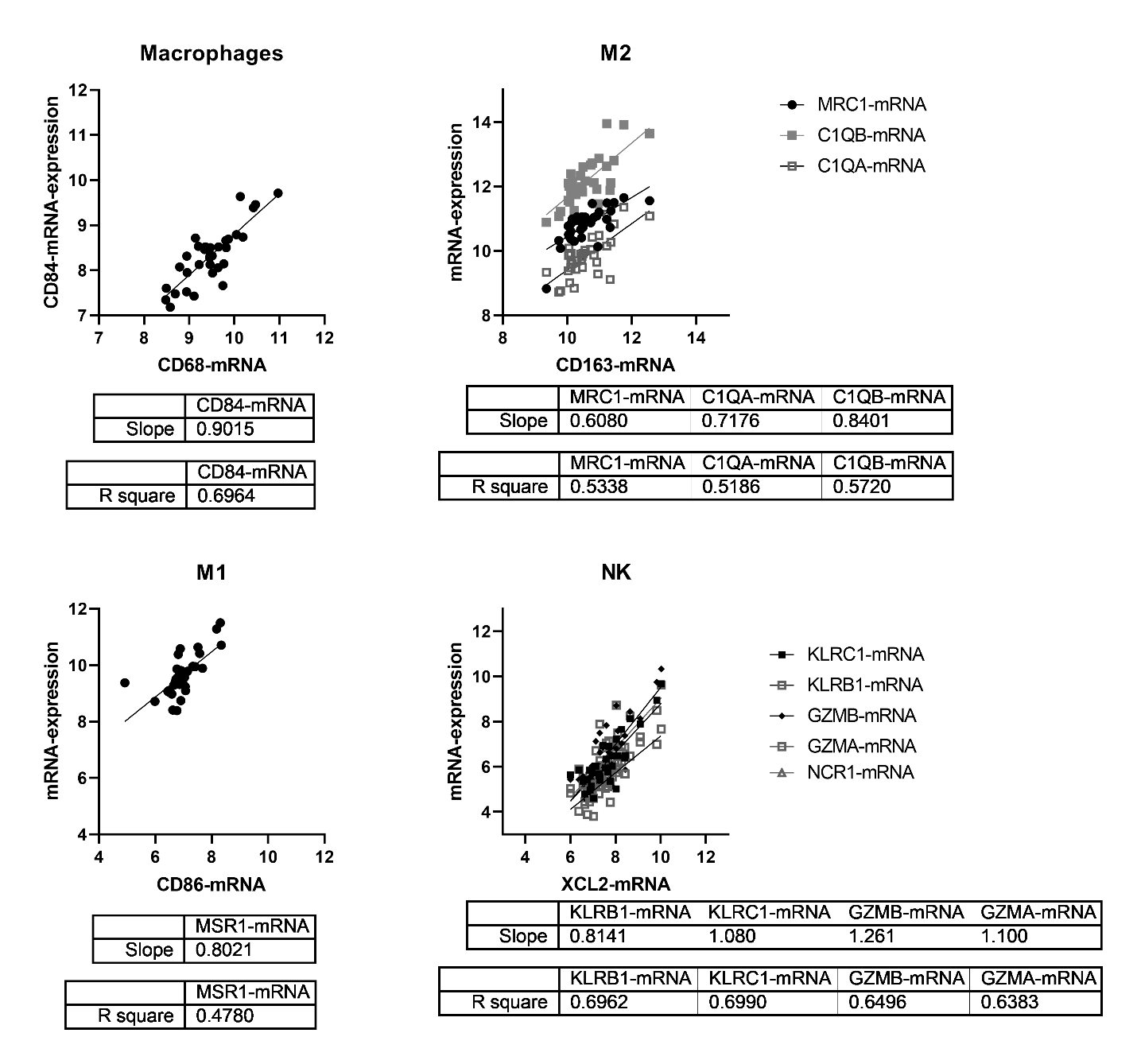
**

**Figure S2. Raw positive control gene counts of the gene expression analysis.** Three healthy samples (red squares) were excluded from the gene expression analysis due to low binding density as depicted by low positive control gene counts compared to the rest of the samples (black circles).

**Figure S3. Betamethasone does not alter placental gene expression.** The effect of betamethasone on fetal outcome is most effective if delivery takes place in the first 48 hours after the first dose, and is absent if the interval is >1 week.(50) The placentas of women with a short (<8 days) compared to a long interval (>8 days) between betamethasone receival and the moment of giving birth did not display any differentially expressed genes **(A)**. The TLR **(B)** and complement scores **(C)** did also not correlate with the interval between betamethasone (beta) receival and birth.


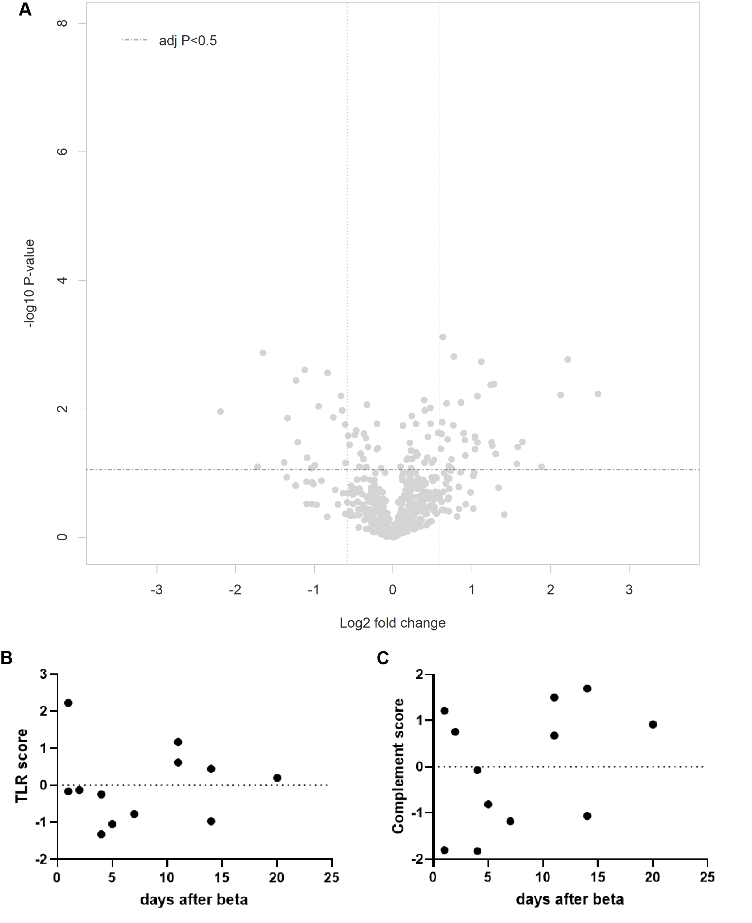

Supplement: Supplementary file 1 [file DataSheet_1.docx]
